# The Inconsistent Assessment of Quality of Life in Patients Treated for Head and Neck Cancer with Anti-EGFR Inhibitors: A Systematic Scoping Review

**DOI:** 10.3390/cancers15092475

**Published:** 2023-04-26

**Authors:** Sarah R. Sutton, April N. Taniguchi, Shaun A. Nguyen, William G. Albergotti, John M. Kaczmar, Alexandra E. Kejner, Jason G. Newman

**Affiliations:** 1 Department of Otolaryngology—Head and Neck Surgery, Medical University of South Carolina, Charleston, SC 29425, USA; 2 School of Medicine, University of Nevada, Reno, NV 89557, USA; 3 Division of Hematology and Oncology, Medical University of South Carolina, Charleston, SC 29425, USA

**Keywords:** anti-EGFR inhibitors, head and neck cancer, quality of life

## Abstract

**Simple Summary:**

There is a correlation between quality of life (QoL) scores and treatment outcomes in patients receiving head and neck cancer (HNC) treatment. Higher QoL scores have been associated with improved survival yet there are considerable differences in the assessment of QoL in clinical trials. The aim of this systematic scoping review is to evaluate the variability of QoL reporting in clinical trials investigating anti-EGFR treatment. Our study confirms no standard method for reporting QoL data in clinical trials for HNC patients. QoL benchmarks are assessed and reported differently between studies. Therefore, these metrics are difficult to evaluate on a larger scale, preventing quantitative analysis. This review identifies the need to standardize the method for QoL assessment.

**Abstract:**

In patients receiving treatment for head and neck cancer (HNC), there is a correlation between quality of life (QoL) scores and treatment outcomes. Higher QoL scores have been associated with improved survival. Despite this, the assessment of QoL in clinical trials varies considerably. Three databases (Scopus, PubMed, and Cinahl) were queried for articles published in English between 2006 and 2022. Two reviewers (SRS and ANT) performed study screening, data extraction, and risk of bias assessment. The authors identified 21 articles that met the inclusion criteria. A total of 5961 patients were evaluated. QoL was reported as average scores for specific variables across five different surveys in 12 included articles. Supplemental QoL data were available in 10 included studies. Critical appraisal of studies indicated a high risk of bias due to the inclusion of trials. There is no standard method for reporting QoL data in clinical trials for HNC patients undergoing treatment with anti-EGFR inhibitors. Future clinical trials should standardize their method for assessing and reporting quality-of-life data to increase patient-centered care and refine treatment choices to optimize survival.

## 1. Introduction

As one of the most common forms of cancer in the United States [1], head and neck cancer (HNC) is a debilitating disease with detrimental effects on patients, both functionally and emotionally. The clinical trials that have transformed the treatment of HNC often provide detailed survival-based outcomes [2,3], yet their assessment of quality of life (QoL) does not appear to be standardized [4,5].

This poses a problem for all HNC treatment modalities, especially for anti-epidermal growth factor receptors (EGFR) monoclonal antibodies such as cetuximab [6,7,8]. Despite their efficacy [9], these medications have significant side effects that negatively impact patient QoL and are associated with toxicities including an acneiform rash, nausea, dyspnea, and fatigue [10,11]. Cetuximab is less toxic than cisplatin, which was the basis for the De-ESCALaTE HPV and RTOG 1016 trials [2,12]. Both of these trials concluded that there was no improvement in toxicity compared with cisplatin [2,12]. Additionally, the intensity of adverse reactions from anti-EGFR medications has been shown to influence QoL and treatment adherence [13,14], yet the assessment of these effects is not consistent in clinical trials.

Trials that assess HNC treatment with anti-EGFR inhibitors demonstrate variable results on their QoL impact. Most trials note that the QoL in patients undergoing treatment for HNC decreases significantly during chemoradiation therapy [2,15,16,17,18,19] but improves by the end of the trial follow-up period. Based on this transient decrease and the inconsistent reporting in studies, it appears that QoL has not been thoroughly investigated. Multimodal treatment regimens for HNC using anti-EGFR inhibitors typically lasts for 6–7 weeks [20], and any reduction in QoL can be difficult for patients to endure, affecting treatment adherence.

The initial objective of this study was to evaluate the impact of anti-EGFR medications on QoL. However, after performing a systematic review, it was impossible to quantitatively assess QoL data due to significant variation in the available, reported data. Appropriate QoL assessment predicts treatment success and decreased morbidity and mortality [21], yet it is not assessed uniformly in the clinical trials for anti-EGFR treatments. Therefore, it is necessary to evaluate QoL changes to provide patient-centered care and ensure optimal adherence to treatment. This systematic scoping review aims to update the scientific community on the variability of QoL reporting in clinical trials investigating anti-EGFR treatment for HNC. A secondary aim is to highlight the importance of addressing QoL concerns while patients are actively being treated.

## 2. Methods

**Figure 1:**
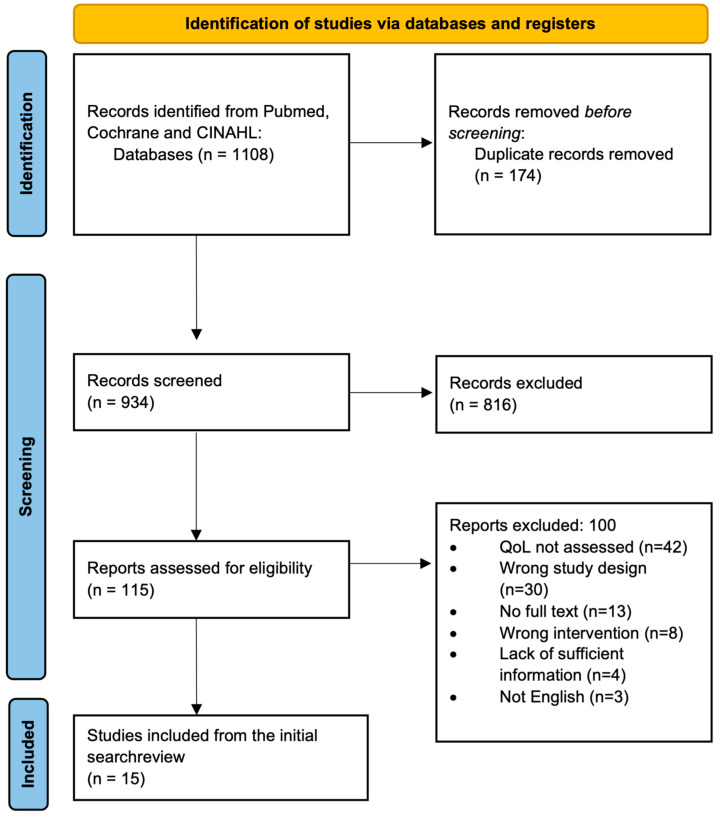
PRIMSA flow chart of study selection.

A systematic scoping review methodology was chosen due to its strengths in summarizing and disseminating findings on a broad topic [22]. The review protocol was not registered or published in peer-reviewed publications. The review followed the five-stage framework outlined by Arksey and O’Malley (2005) and the Preferred Reporting Items for Systematic Reviews and Meta-Analyses Extension for Scoping Reviews (PRIMSA-ScR; see Figure 1) [23].

### 2.1. Stage 1: Identify the Research Question

This review sought to answer the following research questions: What is the variability of QoL reporting in clinical trials investigating anti-EGFR treatment for HNC? Moreover, what is the importance of addressing QoL concerns while patients are actively being treated?

### 2.2. Stage 2: Identify Relevant Literature

An academic librarian assisted in developing the search strategy, which was executed by the two primary authors, SRS and ANT. A systematic review was performed in accordance with PRISMA guidelines [23] using the PubMed, Scopus (Elsevier), and CINAHL (EBSCO) databases from the date of inception to August 2022. The search aimed to identify all English articles related to head and neck cancer treated with anti-EGFR medications. Another goal of the search was to identify articles that assessed these treatments using QoL instruments. Articles written in English were chosen to identify studies and QoL instruments in a widely applicable and universal language, which also avoids validation uncertainty, cross-cultural differences, and inaccuracies in translation. QOL instruments are also primarily developed in English before being translated into other languages [24]. The initial search was made for PubMed and utilized Medical Subject Headings [MESH] and text word [tw] and title and abstract [tiab]. This search was then modified for use in the other databases (Scopus and CINAHL). Key terms for this search were related to head and neck cancer, quality of life evaluation, and the antineoplastic use of EGFR inhibitors such as cetuximab.

### 2.3. Stage 3: Study Selection

At least two authors (SRS and ANT) independently screened records by title and abstract, then by full text using Covidence, a review management software. Disagreements were resolved with all authors. The inclusion criteria required included articles to be full-text, primary studies, English-language, and published in a peer-reviewed journal. Other aspects of the inclusion criteria were as follows: studies needed to evaluate using an anti-EGFR inhibitor within the clinical trial. The population evaluated had head and neck carcinoma. Each included study needed to directly evaluate patient QoL using a validated assessment tool. Included assessment tools included: EORTC QLQ-30, QLQ-H&N-35, FHNSI-10, FACT H&N, VF, HNQOL, and UW-QOL. The full names and characteristics of these surveys are detailed below. All patients were greater than or equal to the age of 18 years old and all studies were published in English. Articles were excluded if they were not specific to head and neck cancer treatment, or if the wrong intervention was assessed. For example, if the immunotherapy evaluated was not anti-EGFR specifically. Articles were also excluded if the study failed to directly evaluate QOL using a validated survey. Additionally, articles were eliminated if they were non-human studies, or had the wrong study design (i.e., case reports, case series, and review articles). Studies that evaluated endocrine neoplasms were also excluded.

#### 2.3.1. Procedure

##### Search Process

This review was conducted in compliance with PRISMA-ScR scoping review methods [23]. The initial search yielded 1108 articles which were uploaded into Covidence, a review management software, for screening. There were 174 duplicate articles. After duplicates were removed, 934 titles and abstracts were screened for relevance independently by two reviewers (SRS and ANT). Authors included titles and abstracts if they mentioned quality of life, if the authors felt there was a high likelihood that quality of life would be mentioned in the full text. There were 816 articles excluded at this stage.

A total of 115 full texts were assessed for eligibility by the same reviewers (SRS and ANT). Articles were included for synthesis following full-text review if they had an appropriate study design, they evaluated the use of anti-EGFR inhibitors, and they provided sufficient information on QoL assessment during the trial so that the data could be utilized to compare to other studies during the synthesis stage. For example, if a study mentioned QoL early in the manuscript but did not report a method for collecting these data or QoL-specific results, they were excluded under this criterion. There were 42 references that were excluded at the full-text stage because quality of life was not assessed in the manuscript. Thirty references had the wrong study design and therefore were excluded. A total of 13 title and abstract entries did not have associated full texts, 8 articles had the wrong intervention, and 4 articles needed to provide more information on QoL to be compared to the other manuscripts. There were 8 articles that assessed the wrong intervention, where anti-EGFR inhibitors were not assessed, and 3 studies were not written in the English language. Following a full-text review, 15 articles met the inclusion criteria and were selected for synthesis. Each study’s references were screened, and 6 additional articles were included, leading to 21 articles. Several articles were published using data from the same trial. To prevent duplication, the first article published was included with the remaining articles excluded. The PRISMA flow diagram is outlined in Figure 1.

### 2.4. Stage 4: Charting the Data

#### 2.4.1. Data Extraction

For the 21 included studies, general information was collected, including author, year of publication, country of study, sample size, study aims, and study design. Data extraction was performed independently by two reviewers (SRS and ANT) and conflicts were resolved by a third reviewer (SAN). The methodology for QoL assessment was collected. This included the validated surveys used, assessment frequency during the treatment period (i.e., at what time points QoL was assessed during the trial and follow-up), patient survey response rate, presentation of QoL assessment (graph, table, both), and whether supplementary data on QoL was available. The supplementary data were collected to assess the reporting of raw QoL data, as mean QoL scores were often presented in the text of included studies.

#### 2.4.2. Level of Evidence and Risk of Bias

References were exported into the review management software (Covidence; Veritas Health Innovation) for study selection. The level of evidence was assessed independently by the two reviewers (SRS and ANT). Conflicts related to the level of evidence for each article were first resolved by a discussion between the two reviewers (SRS and ANT) and then by a third reviewer (SAN) when necessary. Articles were critically appraised to assess the level of evidence per the criteria of the Oxford Center for Evidence-Based Medicine [25]. The risk of bias in each included article was assessed according to the Cochrane Handbook for Systematic Reviews of Interventions (version 6.2) [26].  Two authors (SRS and ANT) performed a pilot assessment on 3 studies to check for consistency of assessment and then performed independent risk assessments on the remaining studies. All disagreements were resolved once both authors came to a consensus. The risk of bias items for randomized trials included random sequence generation, allocation concealment, blinding of participants and personnel, blinding of outcomes assessment, incomplete outcome data, selective reporting, and other bias. The risk of bias for each aspect was graded as low, unclear, or high. Articles were not excluded based on quality since this is not required within the Arksey and O’Malley (2005) framework.

### 2.5. Stage 5: Collating, Summarizing, and Reporting Results

Review results are presented through descriptive statistics of included studies and a narrative summary of findings. Implications of the analysis are then discussed.

## 3. Results

### 3.1. Study Characteristics

**Figure 2:**
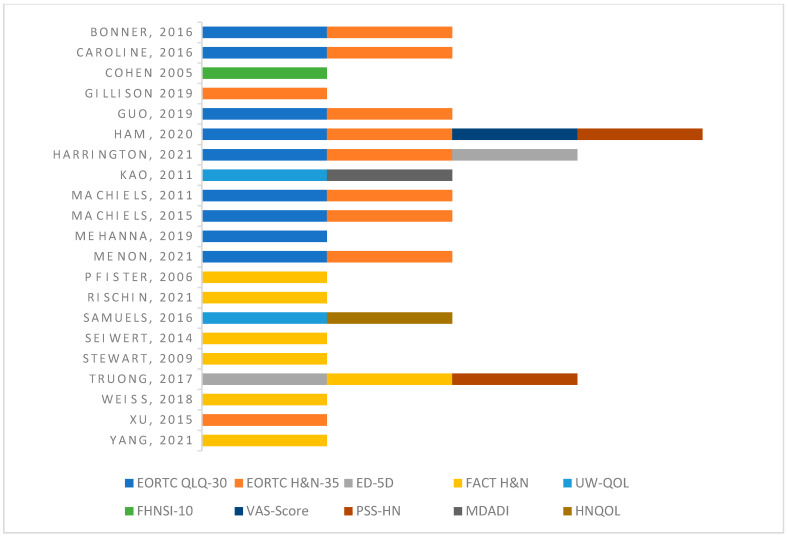
QoL assessment tool used per study [2,3,12,15,16,17,18,19,27,28,29,30,31,32,33,34,35,36,37,38,39].

All 21 studies were clinical trials [2,3,12,15,16,17,18,19,27,28,29,30,31,32,33,34,35,36,37,38,39] that were published between the years 2005 to 2021. Articles selected for inclusion were level 2 studies which included both comparative and randomized clinical studies based on the Oxford Level of Evidence. Critical appraisal of studies indicated a low risk of bias or unclear risk of bias for the majority of included studies (Figures S1 and S2). The potential sources of bias that were most pronounced were in the domains of “blinding of participants and personnel,” “blinding of outcome assessment,” and “other bias.” A list of the included studies, the author, the year, the number of patients evaluated, the treatment modalities evaluated in each study, and the availability of supplemental data are reported in Table 1. The specific surveys that were used in each study, the reporting method for QoL (graph, table, or both), and the timeline of QoL assessment are represented in Table 1 as well as Figure 2.

The most common assessment tool was the European Organization for Research and Treatment of Cancer Quality of Life Head and Neck Module (EORTC QLQ-H&N-35), which was used by 10 studies. The European Organization for Research and Treatment of Cancer Quality of Life (EORTC QLQ-C30) was used by nine studies and the Functional Assessment of Cancer Therapy- Head and Neck (FACT-HN) was used by seven studies. Two studies used the following surveys to evaluate quality of life: The Performance Status Scale for Head and Neck, the University of Washington Quality of Life Questionnaire (UW-QOL), and the EuroQol (EQ-5D). The Functional Assessment of Cancer Therapy Head & Neck Cancer Symptom Index (FHNSI-10), Visual Analog Scale (VAS-scale), Head and Neck Quality of Life Instrument (HNQOL), and MD Anderson Dysphagia Inventory (MDADI) were each used by one article. Of note, the majority of included studies used multiple tools to assess QoL.

### 3.2. Quality of Life Measures

There are several authenticated surveys that are used to assess quality of life in patients undergoing HNC treatment. A brief overview of the most common tools in this review is detailed below. The quality-of-life assessment tools used by each study are represented in Figure 2.

### 3.3. EORTC QLQ-30

The European Organization for Research and Treatment of Cancer (EORTC) developed the QLQ-C30 tool to measure quality of life in cancer patients. The questionnaire measures quality of life with scales in nine categories: three symptom scales (fatigue, pain, and nausea/vomiting), five functional scales (physical, role, cognitive, emotional, and social), and a scale for global health and quality of life. This tool has been a validated measure of quality of life in cancer patients since 1993 [40,41].

### 3.4. QLQ-H&N-35

This assessment tool is used to measure quality of life in head and neck cancer patients exclusively [42,43]. Seven scales are used to assess pain, ability to swallow, taste and smell, speech, social eating, sexuality, and social contact. Problems associated with dentition, dry mouth, mouth-opening, saliva thickness, coughing, and feeling ill are also assessed. It has been validated for use in conjunction with the QLQ-30 [44].

### 3.5. UW-QOLR

The University of Washington Quality of Life Questionnaire (UW-QOL) is a self-administered form for patients who have undergone treatment for head and neck cancer. The different domains include pain, appearance, activity, swallowing, and speech, among others. Patients also rate the importance of each domain. This tool measures the global quality of life in head and neck cancer patients [45].

### 3.6. FACT-HN

The Functional Assessment of Cancer Therapy—Head and Neck (FACT-HN) is a survey that consists of 28 general and 11 head and neck-related topics scored using a Likert-type scale. The themes assessed are physical well-being, social and family well-being, relationship with their physician, emotional well-being, functional well-being, and head and neck-related symptoms [46,47,48].

### 3.7. Timeline of QOL Assessment during Treatment

Each included study assessed the quality of life at varying times based on their specific treatment protocol. Most studies assessed QOL at baseline, and periodically during treatment and in the months following treatment [2,3,12,15,16,17,18,19,27,28,30,31,34,35,38,39]. The exact timeline for assessing QOL for each study is outlined in Table 1.

## 4. Discussion

HNC is a profound disease that affects approximately 900,000 individuals annually and causes roughly 400,000 deaths per year [1]. Fortunately, survival outcomes are improving as treatments continue to advance [49]. QoL assessment is a patient-reported outcome (PRO) that is utilized to gauge the impact of cancer and treatment on a patient’s daily life. It evaluates a patient’s overall well-being and contentment while they battle to survive both their disease and treatment. Therefore, it is an integral evaluation for clinical trials that assess therapeutic agents with negative side effects, such as anti-EGFR inhibitors. Additionally, previous studies have identified QoL as an independent predictor for overall survival, regardless of cancer staging [50]. Given the clinical implications of these findings, this scoping review was conducted to identify current gaps in QoL appraisal and to highlight the importance of standardizing this assessment in clinical trials.

The results of this review identify both limited and varied QoL data in the included clinical trials. A total of 11 different PRO surveys were utilized among the 21 studies to assess the QoL and functional status of the patients. The most common assessment was the QLQ-30 combined with the H&N-35, which was seen in nine studies. However, even in those nine studies, variation in the timeline of assessment as well as the reporting method was seen. For example, the clinical trial conducted by Bonner et al. [19] used these two surveys at baseline, week 4, week 8, month 8, and month 12. Their QoL results were reported using both graphs and tables. Using the same tools, the Caroline study [27] evaluated QoL at baseline, when the patient completed radiotherapy, and again at 6 weeks and 24 weeks post-radiotherapy. This study did not report quality of life data using a graph or table and provided no supplemental data. Therefore, QoL data could not be compared between these two studies despite the use of the same modalities. This is just one example of the variation found in the included studies. 

The most common tools were the QLQ-30 and/or H&N-35, used in approximately 52% of studies, and the FACT-H&N, used in 33% of the included articles. There is currently no method to assess how the results of these surveys compare to each other, prohibiting analysis on a larger scale [51]. Additionally, one included article only assessed quality of life using the H&N-35, which is not considered a validated assessment tool unless used with QLQ-30 [42]. These findings illustrate the incoherent method of QoL data collection and reporting. 

Given the improvements seen in survival outcomes [49], the current paradigm for successful treatment needs to expand and include patient-centered outcomes. The justification for this change is exhibited by a study that examined different preferences for quality of life versus the longevity of life (LoL) in HNC treatment [52]. Younger HNC patients selected aggressive treatment to increase LoL whereas older patients preferred treatments to improve QoL. A similar study found that QoL has a direct impact on treatment adherence in patients with gastrointestinal carcinomas [53]. These findings convey that patients are driven by different motivators, demonstrating the need for patient-centered care when selecting and monitoring appropriate treatment regimens. Treatment with anti-EGFR inhibitors is associated with severe side effects, and the patient’s ability to tolerate them should be balanced with survival goals. 

Additionally, although not for HNC specifically, QoL assessment during brain cancer treatment has been identified as a tool that improves the physician–patient relationship and patient communication [54]. The findings of this study serve as a reminder that measuring QoL outcomes can lead to PRO improvement by simply administering the survey. This discovery provides additional incentive for why QoL should be assessed during oncological treatment for head and neck tumors. 

This scoping review identified multiple studies that reported a negative impact on QoL using anti-EGFR inhibitors [2,15,16,17,18,19]. Yet, these findings appear to be overlooked as they are transient and are not prominent beyond several months after treatment. Active treatment usually spans 6–7 weeks [20], and this period is distressing for patients. Despite improvement in QoL after treatment, research indicates that patient care and comfort can be improved with increased attention to PROs during active treatment [54]. Significant negative impacts, even when limited to active treatment, should not be overlooked. This is an area where current clinical trials in this field have room to improve. 

There is a myriad of challenges that prohibit the standardized use of QoL assessment tools in clinical trials. These obstacles are derived from the subjective and variable nature of QoL reporting. It is difficult to evaluate QoL with accurate measurements and make inter-patient comparisons. Furthermore, there is no data to confirm which validated survey is the best to use nor is there evidence on how to compare scores of one tool to those of another [55]. This creates uncertainty regarding survey selection and the most appropriate timeline for QoL assessment in clinical trial designs [56].

In this review, the clinical trials utilized different surveys for QoL, and some only reported their significant survey findings which were different among the studies [19,30]. If all outcomes regardless of significance were reported, the QoL data could have been pooled to identify which QoL outcomes were most affected and how those categories could be better addressed in the future. Most of the studies identified in this scoping review utilize multiple QoL assessment tools for a holistic appraisal. This strategy creates respondent fatigue for patients, which has been shown to increase the amount of missing data and poor reporting of PROs in clinical trials [57]. This is an additional complication in the assessment of QoL for clinical trials. 

A notable strength of this review was the comprehensive approach to EGFR inhibitors and QoL measurements. The thorough search strategy allowed us to be reasonably confident in capturing all relevant QoL instruments and studies in the English language. This study is important in evaluating the most current literature on QoL assessment in HNC but has its own limitations. This scoping review was limited to articles that evaluated anti-EGFR inhibitors and does not evaluate QoL for other commonly utilized medications such as PD-1 inhibitors. Another limitation of our study is the repetition of multiple studies analyzing the same results from one clinical trial. To avoid duplication of PROs, only the first published article from each trial was included in our review. It is possible that other studies related to the same clinical trial assessed quality of life more thoroughly. Although the aim of this scoping review was to assess all articles that reported cetuximab, this created some additional restrictions. Due to the nature of a scoping review, some included articles evaluated cetuximab treatment for metastatic or recurrent disease. In this scenario, the impact of anti-EGFR treatments on quality of life is confounded by other therapies. Additionally, it is difficult to pool QoL scores when the treatment modalities are not exactly the same. This was another limitation as the majority of included studies evaluated anti-EGFR inhibitors in conjunction with other treatment modalities. Finally, a limitation of this review is that articles in the grey literature or non-English articles and QoL instruments were excluded. The reasons for this are described earlier. However, we are aware of non-English manuscripts and QoL instruments that may exist. 

The initial intention of this study was to perform a meta-analysis of the QoL scores among patients undergoing HNC treatment with anti-EGFR inhibitors. This antineoplastic agent was chosen for its debilitating side effect profile [58,59,60]. It was found that studies utilizing the same assessment tools differed in their reporting methods, and this prevented doing a pooled analysis. This reiterates a similar conclusion made in 2001 [5] and 2016 by Rogers et al. who identified the need for a strict standard for QoL assessment and reporting in clinical trials [4]. When considering the inconsistent nature of the assessment, it is concerning that progress has not been made in the past twenty years and suggests that little is known about the impact of novel cancer treatments on QoL at a population level. A standardized evaluation will facilitate a patient-centered approach to therapy and ultimately improve therapeutic success. Future directions of this research should aim to assess the QoL assessment methods in other treatment modalities for head and neck cancer such as PD-1 inhibitors.

## 5. Conclusions

The findings of this systematic scoping review restate the urgency to standardize the reporting system for QoL in HNC clinical trials. Important gaps in knowledge have been identified. Current HNC-specific QoL instruments are few. Objective appraisal criteria for measurement properties and clinical and pragmatic considerations are required to recommend the best QoL instruments. It is crucial to overcome the presented challenges associated with the validated surveys and collectively decide how to utilize these tools best as a field. Uniform QOL data will convey any important findings that are found from different treatment options and allow clinicians to implement a more patient-centered approach to treatment decisions in HNC.

## Figures and Tables

**Table 1:** Prevalence of quality-of-life data reported per article.

Study Author, Year	Number of Patients	Oxford Level of Evidence	Clinical Trial Treatment Modalities	Verified QoL Assessment Tool	Method for Reporting QOL Data (Graph, Table, Both)	Timeline of QOL Assessment	Supplemental Data Provided (Yes/No)
Bonner, 2016 [19]	168	II	Cetuximab and radiotherapy vs. radiotherapy alone	EORTC QLQ-30, QLQ-H&N-35	Both	Baseline, wk 4, wk 8, month 8, month 12	Yes
Caroline, 2016 [27]	20	II	Cetuximab plus radiotherapy versus concomitant cisplatin plus radiotherapy	EORTC QLQ-30, QLQ-H&N-35	Neither	Baseline, last day of radiotherapy, 6 wks, 24 wks after radiotherapy	No
Cohen, 2005 [28]	70	II	Gefitinib daily in patients with recurrent or metastatic SCCHN	FHNSI-10	Graph	Baseline, day 7,14,21,28 of cycle 1 and day 28 of every cycle after that	No
Gillison, 2019 [12]	805	II	Radiotherapy plus cetuximab in HPV+ oropharyngeal cancer	EORTC QLQ-H&N-35	Neither	Baseline, end of treatment, 3,6,12 months	No
Guo, 2019 [29]	340	II	Afatinib vs. methotrexate after failed platinum-based therapy	EORTC QLQ-30, QLQ-H&N-35	Both	Not outlined	Yes
Ham, 2020 [30]	45	II	Methotrexate with or without cetuximab for recurrent or metastatic SCCHN	EORTC QLQ-30, QLQ-H&N-35, PSS-HN, VAS-Score	Table	Baseline, wk 8, “progressive disease”	Yes
Harrington, 2021 [31]	495	II	Pembrolizumab alone or with chemotherapy vs. cetuximab with chemotherapy for recurrent or metastatic SCCHN	EORTC QLQ-30, QLQ-H&N35, EQ-5D	Both	Baseline, wk3, wk6, wk9, q 6 wks for 1 year	Yes
Kao, 2011 [15]	33	II	Concurrent 5-FU, hydroxyurea, cetuximab, and hyperfractionated intensity-modulated RT for locally advanced HNC	UW-QOLR, MDADI	Graph	Baseline, after chemo, wk 4, month 4, month 10, month 12	No
Machiels, 2011 [32]	286	II	Zalutumumab vs. supportive care alone in patients with recurrent or metastatic SCCHN	EORTC QLQ-30, QLQ-H&N-35	Neither	Not definitively outlined. Assessed at wk 8 and wk 16	No
Machiels, 2015 [33]	483	II	Afatinib vs. methotrexate in patients with recurrent or metastatic SCCHN	EORTC QLQ-C30, QLQ H&N-35	Graph	Not outlined	No
Mehanna, 2019 [2]	334	II	Radiotherapy plus cisplatin or cetuximab in low-risk HPV+ oropharyngeal cancer	EORTC QLQ-30	Graph	Baseline, radiotherapy completion, month 3, month 6, month 12, month 24	No
Menon, 2021 [34]	536	II	Concurrent chemoradiation with cisplatin and nimotuzumab vs. cisplatin alone in locally advanced HNC	EORTC QLQ-30, QLQ-H&N-35	Both	Baseline, 3,6,12,18,24,36 months	Yes
Pfister, 2006 [35]	21	II	Concurrent cetuximab, cisplatin, and concomitant boost radiotherapy for locoregionally advanced SCCHN	FACT H&N	Neither	Wk 6, wk 4–6 f/u, 12–16 wk f/u	No
Rischin, 2021 [3]	200	II	Radiation therapy with weekly cisplatin or cetuximab in low-risk HPV-associated oropharyngeal cancer	FACT-H&N	Neither	Baseline, week 9 post-RT, 6,12,24 months	No
Samuels, 2016 [16]	53	II	Radiation therapy with cetuximab compared to chemotherapy and radiation therapy in patients with stage III/IV HPV+ OPC	HNQOL, UW-QOL	Table	Pretreatment, month 3, month 12,	Yes
Seiwert, 2014 [36]	124	II	Afatinib vs. cetuximab in metastatic or recurrent SCCHN	EORTC QLQ-C30, QLQ H&N-35	Neither	Not outlined	No
Stewart, 2009 [37]	486	II	Gefitinib vs. IV methotrexate for recurrent SCCHN	FACT-HN	Neither	Not outlined	No
Truong, 2017 [38]	891	II	Concurrent accelerated radiation plus cisplatin with or without cetuximab for locally advanced HNC	FACT-HN, EQ-5D, PSS-HN	Table	Pretreatment, 1 year, 5 years	Yes
Weiss, 2018 [17]	40	II	Induction chemotherapy with carboplatin, nabpaclitaxel, and cetuximab for at least N2b nodal status or surgically unresectable SCCHN	FACT-HN	Figure	Pretreatment, treatment break, 1 year f/u	Yes
Xu, 2015 [18]	44	II	Weekly cetuximab concurrent with IMRT in locally advanced nasopharyngeal carcinoma	QLQ-H&N-35	Table	Every 3 months for 2 years	Yes
Yang, 2021 [39]	126	II	Cetuzimab combined with IMRT and concurrent chemotherapy in locally advanced nasopharyngeal carcinoma	FACT-HN	Neither	Pretreatment, 3 months after treatment	No

SCCHN—Squamous cell carcinoma of the head and neck; IMRT—intensity modulated radiation therapy; OPC—oropharyngeal cancer.

## Data Availability

No new data were created for this study. Additional information will be provided upon reasonable request to the corresponding author.

## Supplementary Materials

The following supporting information can be downloaded at: https://www.mdpi.com/article/10.3390/cancers15092475/s1, Risk of bias assessment graphs are included as supplementary materials. Figure S1: Risk of bias summary: review authors’ judgments about each risk of bias item for each included study. Figure S2: Risk of bias graph: review authors’ decisions about each risk of bias item presented as percentages across all included studies.
