# Supplementary material for: The Inconsistent Assessment of Quality of Life in Patients Treated for Head and Neck Cancer with Anti-EGFR Inhibitors: A Systematic Scoping Review
Source: Cancers (Basel). 2023 Apr 26;15(9):2475. doi: 10.3390/cancers15092475 (PMC10177329; doi:10.3390/cancers15092475)

Figure S1: Risk of bias summary: review authors' judgements about each risk of bias item for each included study.

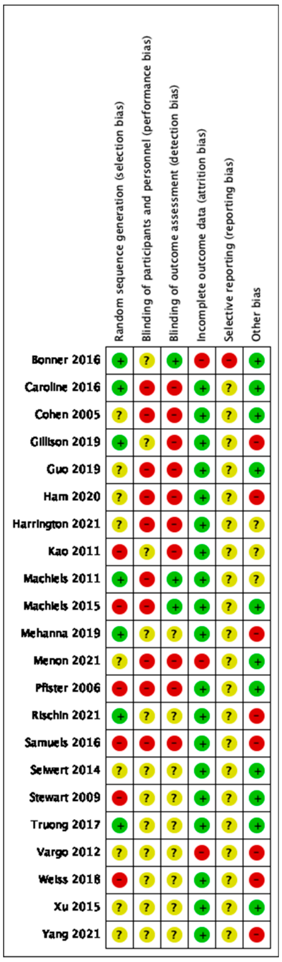

Figure S2: Risk of bias graph: review authors' judgements about each risk of bias item presented as percentages across all included studies.

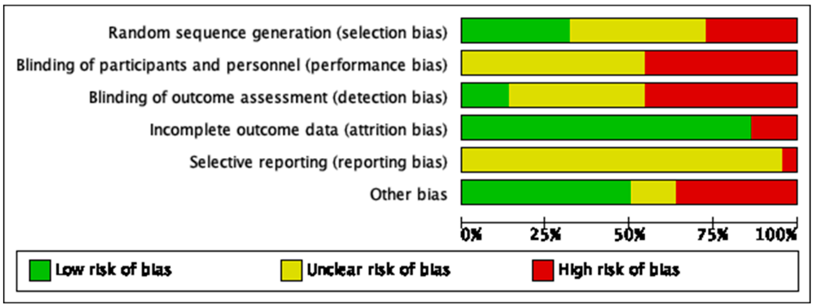

Supplement: Supplementary file 1 [file cancers-15-02475-s001.zip › cancers-2296584-supplementary.pdf]
